# 司坦唑醇和达那唑治疗非重型再生障碍性贫血的疗效及其对CD4^+^CD25^+^Foxp3^+^调节性T细胞的影响

**DOI:** 10.3760/cma.j.issn.0253-2727.2022.02.013

**Published:** 2022-02

**Authors:** 红敏 李, 章彪 龙, 涛 王, 冰 韩

**Affiliations:** 1 中国医学科学院、北京协和医学院北京协和医院血液科，北京 100730 Department of Hematology, Peking Union Medical College Hospital, Chinese Academy of Medical Sciences and Peking Union Medical College, Beijing 100730, China; 2 中国医学科学院、北京协和医学院基础医学研究所，北京 100730 Institute of Basic Medical Sciences, Chinese Academy of Medical Sciences and Peking Union Medical College, Beijing 100730, China

再生障碍性贫血（AA）是一种骨髓造血衰竭性疾病，可分为重型AA（SAA）和非重型AA（NSAA）[1]–[2]。NSAA患者目前尚无一致认可的治疗方案。由于部分NSAA患者可进展为SAA或发生克隆演变，目前国内外学者多提倡给予早期干预[3]。达那唑是人工合成的雄激素类药物，文献报道其单药或联合环孢素A（CsA）治疗AA均有一定疗效，可能具有免疫调节作用[4]–[8]。司坦唑醇是我国应用广泛的雄激素类药物，目前国际上尚无司坦唑醇治疗AA的临床应用数据。本研究通过分析于我中心采用司坦唑醇或达那唑联合CsA治疗的获得性NSAA患者临床资料，比较司坦唑醇和达那唑在治疗NSAA的疗效及安全性，同时采用流式细胞术检测患者治疗前后CD4^+^CD25^+^Foxp3^+^调节性T细胞（Treg）水平，观察不同治疗方案对患者Treg的影响。

## 病例与方法

1. 病例：回顾性分析自2017年1月至2019年1月于北京协和医院门诊治疗的获得性NSAA患者临床资料，排除至随访截止日期治疗时间不足半年的患者。AA诊断符合《再生障碍性贫血诊断治疗专家共识（2017年版）》[9]标准，严重程度按照Camitta标准[10]判定。PNH克隆阳性定义为流式细胞术CD235a^+^CD55^−^/CD235a^+^CD59^−^红细胞比例>5％或CD24^−^/Flear^−^粒细胞比例>5％（10 000个有核细胞）。本研究经北京协和医院伦理委员会批准。

2. 治疗：根据治疗方案不同将患者分为3组：① CsA单药组：CsA 3～5 mg·kg^−1^·d^−1^，分两次间隔12 h口服，维持CsA血药浓度150～250 µg/L。② CsA联合司坦唑醇组：司坦唑醇 2 mg，每日3次口服；CsA治疗剂量同上。③ CsA联合达那唑组：达那唑 200 mg，每日3次口服；CsA治疗剂量同上。

3. 评价标准：AA治疗反应评价标准参考《2015英国血液学标准委员会指南》[11]，包括完全治疗反应（CR）、部分治疗反应（PR）、无治疗反应（NR），CR和PR为有效（OR）。不良反应分级参考国际通用不良反应分级（CTCAE）3.0版。伴有血清丙氨酸转氨酶（ALT）或血清总胆红素（TBIL）增高者被认为存在肝功能异常。ALT增高程度定义：轻度，40 U/L～≤100 U/L；中度，100 U/L～≤200 U/L；重度，ALT>200 U/L。TBIL增高程度定义：轻度，17.1 µmol/L～≤34.2 µmol/L；中度，34.2 µmol/L～≤51.3 µmol/L；重度，TBIL>51.3 µmol/L。肾功能不全程度以肾小球滤过率（GFR）表示：轻度，≥60 ml/min/1.73m^2^～90 ml/min/1.73m^2^；中度，≥30 ml/min/1.73m^2^～60 ml/min/1.73m^2^；重度，GFR<30 ml/min/1.73m^2^。

4. 流式细胞术检测患者外周血Treg水平：收集患者治疗前及治疗后6个月新鲜抗凝静脉血2 ml，流式细胞术检测CD4^+^CD25^+^Foxp3^+^ Treg比例，比较不同方案治疗前后Treg水平变化，并收集6例健康志愿者静脉血标本作为正常对照。

5. 统计学处理：采用SPSS 22.0软件进行数据描述及统计分析。符合正态分布的计量资料采用均数±标准差表示，组间比较采用*t*检验或方差分析。不符合正态分布的计量资料采用中位数（范围）表示，组间比较采用非参数检验。计数资料采用百分比表示，不同治疗方案疗效比较采用Pearson卡方检验或Fisher精确概率法，组内两两比较采用bofferoni校正。治疗前后患者Treg比例比较采用配对秩和检验。

## 结果

1. 患者基线资料：本研究共纳入74例NSAA患者，其中男32例（43.2％），女42例（56.8％），中位年龄40（14～68）岁。CsA联合司坦唑醇治疗组40例（54.1％），CsA联合达那唑治疗组14例（18.9％），CsA单药治疗组20例（27.0％）。各组患者基线特征及随访时间差异均无统计学意义（*P*>0.05）（表1）。

**表1:** 三种不同方案治疗组获得性NSAA患者基线特征及随访时间比较

变量	CsA+司坦唑醇（40例）	CsA+达那唑（14例）	CsA单药（20例）	*P*值
性别［例（％）］				0.357
男	20（50.0）	4（28.6）	8（40.0）	
女	20（50.0）	10（71.4）	12（60.0）	
年龄［岁，*M*（范围）］	41（14～67）	42.5（15～68）	45.5（38～61）	0.524
初诊时外周血细胞计数				
ANC［×10^9^/L，*M*（范围）］	1.2（0.7～3.8）	2.3（0.7～4.8）	1.4（0.3～4.5）	0.153
HGB［g/L，*M*（范围）］	85（52～125）	116（45～152）	93（47～133）	0.144
PLT［×10^9^/L，*M*（范围）］	31（5～229）	27（4～32）	23（6～56）	0.314
Ret［×10^9^/L，*M*（范围）］	36.3（23.1～58.0）	36.8（26.7～51.1）	37.3（20.4～54.9）	0.301
PNH克隆阳性［例（％）］	2（5.0）	0（0）	2（10.0）	0.793
随访时间［月，*M*（范围）］	16.5（9～28）	17（6～20）	15（12～24）	0.832

注：NSAA：非重型再生障碍性贫血；CsA：环孢素A；ANC：中性粒细胞绝对计数；Ret：网织红细胞绝对计数；PNH：阵发性睡眠性血红蛋白尿

2. 治疗反应：中位随访16.5（6～28）个月，74例患者均经历了至少半年的治疗。40例接受CsA联合司坦唑醇治疗的患者中，CR 12例（30.0％），PR 16例（40.0％），OR率为70.0％。14例接受CsA联合达那唑治疗的患者中，CR 4例（28.6％），PR 4例（28.6％），OR率为57.2％。20例接受CsA单药治疗的患者中，CR 2例（10.0％），PR 6例（30.0％），OR率为40.0％。三组患者OR率、CR率两两比较差异均无统计学意义（*P*值均>0.017）。CsA联合司坦唑醇治疗组患者红系治疗反应率为60.0％，高于CsA联合达那唑治疗组（28.6％）（*P*＝0.063>0.017）及CsA单药治疗组（20.0％）（*P*＝0.006）；CsA联合司坦唑醇治疗组与CsA联合达那唑治疗组患者粒系治疗反应相当（55.0％、57.1％，*P*＝1.000），高于CsA单药治疗组的25.0％（*P*＝0.032，*P*＝0.080）；三组血小板治疗反应率分别为45.0％、57.1％、40.0％，两两比较差异均无统计学意义。三种不同治疗方案中位起效时间分别为3（1～8）、2.5（2～6.5）、3.3（1.5～5.5）个月，两两比较差异均无统计学意义，其3个月及6个月OR率分别为45.0％、28.6％、10.0％及65.0％、42.9％、40.0％。

3. 不良反应：最常见的不良反应为肾功能异常（44.6％，33/74），其中仅2例（2.7％）患者因重度肾功能不全而换用其他治疗方案，其余患者经CsA减量后肾功能好转。肝功能异常发生于约20％患者中，其中ALT增高者占16.2％（12/74），胆红素增高者占18.9％（14/74），均为轻中度增高，经对症治疗后好转。其他较少见不良反应包括女性月经紊乱（16.7％）、胃肠道反应（12.2％）、毛发增多（10.8％）、肌痛/肌肉震颤（8.1％）、齿龈增生（5.4％）、痤疮（2.7％）等，经对症治疗或短暂停药后好转，三组患者不良反应比较差异均无统计学意义（表2）。

**表2:** 三种不同方案治疗组获得性NSAA患者不良反应发生率比较［例（％）］

不良反应	CsA+司坦唑醇（40例）	CsA+达那唑（14例）	CsA单药（20例）	*P*值
肾功能不全	18（45.0）	8（57.1）	7（35.0）	0.440
轻	10（25.0）	4（28.6）	4（20.0）	
中	7（17.5）	4（28.6）	2（10.0）	
重	1（2.5）	0（0）	1（5.0）	
ALT增高	8（20.0）	2（14.3）	2（10.0）	0.598
轻	5（12.5）	1（7.1）	1（5.0）	
中	3（7.5）	1（7.1）	1（5.0）	
血清胆红素增高	8（20.0）	4（28.6）	2（10.0）	0.383
轻	6（15.0）	2（14.3）	2（10.0）	
中	2（5.0）	0（0）	0（0）	
女性月经紊乱^a^	3（15.0）	2（20）	1（8.3）	0.733
胃肠道反应	5（12.5）	2（14.3）	2（10.0）	0.927
毛发增多	5（12.5）	2（14.3）	1（5.0）	0.608
肌痛、肌肉震颤	3（7.5）	0（0）	1（5.0）	0.563
齿龈增生	2（5.0）	1（7.1）	1（5.0）	0.950
痤疮	2（5.0）	0（0）	0（0）	0.417
癫痫发作	0（0）	0（0）	1（5.0）	0.230

注：CsA：环孢素A；NSAA：非重型再生障碍性贫血；a：CsA+司坦唑醇、CsA+达那唑、CsA单药治疗组分别有20、10及12例女性患者

4. 流式细胞术检测治疗前后患者外周血Treg比例：74例患者中，我们共获取19例患者治疗前及治疗半年后静脉血标本。其中，CsA+司坦唑醇治疗者12例，CsA+达那唑治疗者4例，CsA单药治疗者3例。同期检测6例健康志愿者Treg/CD4^+^ T细胞比例作为正常对照，其中，男2例，女4例，中位年龄36（26～55）岁。19例患者治疗前Treg/CD4^+^ T细胞比例为2.7％（1.4％～5.0％），显著低于正常对照的4.7％（3.7％～6.3％）（*P*＝0.010）。经过不同方案治疗半年后，10例患者Treg比例增高［1.35％（0.5％～1.9％），*P*<0.001］。其中CR 5例（50.0％），PR 3例（30.0％），OR率为80.0％。9例不伴有Treg比例增加的患者中，CR 2例（22.2％），PR 3例（33.3％），OR率为55.5％。配对比较显示CsA+司坦唑醇治疗能上调NSAA患者Treg细胞水平［2.25％（1.6％～5.0％）对3.45％（1.5％～5.3％），*P*＝0.059］。4例经CsA+达那唑治疗的患者，3例治疗后Treg比例增高［4.0％（2.2％～4.9％）对4.05％（3.3％～5.4％），*P*＝0.715］，而3例经CsA单药治疗的患者均出现了Treg比例下降［2.8％（1.4％～4.5％）对1.8％（1.0％～3.6％），*P*＝0.109］（图1）。

**图1:**
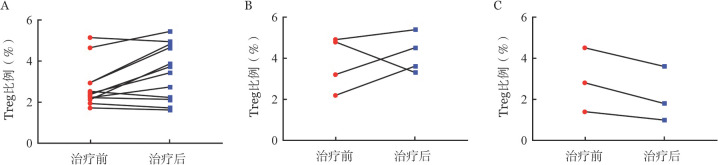
不同方案治疗前后获得性非重型再生障碍性贫血患者调节性T细胞（Treg）水平 A：环孢素A（CsA）联合司坦唑醇治疗组；B：CsA联合达那唑治疗组；C：CsA单药治疗组

## 讨论

早在20世纪中期，雄激素就被用于骨髓衰竭性疾病的促造血治疗。随着我们对AA发病机制的深入了解，免疫抑制治疗已成为AA治疗的基石。近年来，TPO受体激动剂艾曲波帕（Eltrombopag）在促造血方面显示出较好疗效，并逐渐为临床所应用[12]–[14]。尽管如此，由于雄激素类药物价格低廉，易于获取，仍处于临床促造血药物的主流地位。本研究回顾了我中心采用CsA联合司坦唑醇或达那唑以及CsA单药治疗的获得性NSAA患者临床数据，共纳入74例接受至少半年治疗的患者，结果显示CsA联合司坦唑醇或达那唑治疗的患者OR率均高于CsA单药治疗患者，肯定了CsA联合雄激素在NSAA治疗中的积极作用。

关于达那唑在AA中的应用，既往研究有较多报道。一项单中心回顾性研究显示，达那唑单药一线治疗获得性AA的有效率可达46％，CR率27％，5年生存率为41％[4]。另一项研究采用CsA联合达那唑治疗34例NSAA患者，其OR率为58.6％[15]，其他研究也采用CsA+达那唑+ATG±G-CSF的多药联合方案，其有效率为50％～60％[16]–[17]。本研究中，我们采用CsA+达那唑治疗的获得性NSAA患者的OR率为57.2％，与既往研究一致。王书春等[18]报道了采用司坦唑醇治疗的114例儿童NSAA患者，其1年CR率可达13.3％，5年生存率为100.0％。中国医学科学院血液病医院宋琳等[19]采用CsA+司坦唑醇治疗的输血依赖NSAA患者的半年OR率达55.8％，本研究中CsA+司坦唑醇治疗NSAA患者的半年OR率为65.0％，略高于既往报道。值得注意的是，本研究中，虽然相对于CsA+达那唑，CsA联合司坦唑醇治疗NSAA患者OR率差异无统计学意义，但其在促红系造血中却具有优势。提示贫血症状负荷重的NSAA患者可能更能从CsA联合司坦唑醇治疗中获益。因为对于NSAA患者，最常见的主诉为贫血所致乏力症状，也是影响其生活质量的重要因素[20]。

CD8^+^ T细胞异常扩增与活化介导了获得性AA造血干细胞损害[21]，而伴有Foxp3高表达的Treg能够调节T细胞分化并抑制活化CD8^+^ T细胞的细胞毒作用，对于维持T细胞系统正常功能具有重要作用[22]。Shi等[23]研究证实获得性AA患者骨髓及外周血Treg比例均显著下降，且存在功能受损，对细胞毒性T细胞抑制能力减弱。本研究结果显示NSAA患者外周血CD4^+^CD25^+^Foxp3^+^ Treg比例明显低于健康对照者，与上述研究结果一致。同时，治疗后Treg比例增加的患者OR率高于Treg比例未增加者，提示以上调Treg为靶向的治疗或许能增加AA患者获益。由于Treg细胞正常数量及功能的维持依赖持续性IL-2表达，而钙调磷酸酶抑制剂能显著抑制T细胞IL-2表达[23]。目前体内外研究多认为CsA对Treg具有抑制作用[25]–[26]。本研究中，3例采用CsA治疗的NSAA患者均出现Treg细胞比例的下降，在一定程度上支持上述观点。而联合司坦唑醇治疗后，患者Treg比例较治疗前显著增加，支持司坦唑醇对NSAA患者Treg细胞的积极作用。由于样本量限制，本研究中，仅4例采用CsA+达那唑治疗的患者进行了治疗前后Treg检测，其中3例患者出现Treg比例的增高，也在一定程度上支持了达那唑对增加Treg的积极作用。我们推断，联合司坦唑醇和达那唑治疗能弥补CsA对NSAA患者Treg的抑制作用，在发挥免疫抑制作用的同时增加患者免疫耐受能力。

综上，本研究首次比较了司坦唑醇和达那唑在NSAA中的疗效，研究结果提示司坦唑醇在NSAA治疗中具有积极作用，其疗效不亚于达那唑，且在促红系治疗反应中更具优势。获得性NSAA患者外周血Treg水平低于健康对照者，而相较于CsA单药，CsA联合司坦唑醇或达那唑治疗能上调患者Treg水平，提示司坦唑醇和达那唑可能是通过免疫调节效应发挥积极治疗作用。值得注意的是，由于本研究为回顾性研究，患者治疗选择存在一定程度偏倚。且本研究样本数量有限，研究结论尚需进一步前瞻性随机对照临床试验证实。
